# Reproductive Physiology in Young Men Is Cumulatively Affected by FSH-Action Modulating Genetic Variants: *FSHR* -29G/A and c.2039 A/G, *FSHB* -211G/T

**DOI:** 10.1371/journal.pone.0094244

**Published:** 2014-04-09

**Authors:** Marina Grigorova, Margus Punab, Anna Maria Punab, Olev Poolamets, Vladimir Vihljajev, Birutė Žilaitienė, Juris Erenpreiss, Valentinas Matulevičius, Maris Laan

**Affiliations:** 1 Human Molecular Genetics Research Group, Institute of Molecular and Cell Biology, University of Tartu, Tartu, Estonia; 2 Andrology Unit, Tartu University Hospital, Tartu, Estonia; 3 Institute of Endocrinology, Medical Academy, Lithuanian University of Health Sciences, Kaunas, Lithuania; 4 Andrology Laboratory, Riga Stradins University, Riga, Latvia; University of Nevada School of Medicine, United States of America

## Abstract

*Follicle-Stimulating Hormone Receptor* (*FSHR*) -29G/A polymorphism (rs1394205) was reported to modulate gene expression and reproductive parameters in women, but data in men is limited. We aimed to bring evidence to the effect of *FSHR* -29G/A variants in men. In Baltic young male cohort (n = 982; Estonians, Latvians, Lithuanians; aged 20.2±2.0 years), the *FSHR* -29 A-allele was significantly associated with higher serum FSH (linear regression: effect 0.27 IU/L; *P* = 0.0019, resistant to Bonferroni correction for multiple testing) and showed a non-significant trend for association with higher LH (0.19 IU/L) and total testosterone (0.93 nmol/L), but reduced Inhibin B (−7.84 pg/mL) and total testes volume (effect −1.00 mL). Next, we extended the study and tested the effect of *FSHR* gene haplotypes determined by the allelic combination of *FSHR* -29G/A and a well-studied variant c.2039 A/G (Asn680Ser, exon 10). Among the *FSHR* -29A/2039G haplotype carriers (A-Ser; haplotype-based linear regression), this genetic effect was enhanced for FSH (effect 0.40 IU/L), Inhibin B (−16.57 pg/mL) and total testes volume (−2.34 mL). Finally, we estimated the total contribution of three known FSH-action modulating SNPs (*FSHB* -211G/T; *FSHR* -29G/A, c.2039 A/G) to phenotypic variance in reproductive parameters among young men. The major FSH-action modulating SNPs explained together 2.3%, 1.4%, 1.0 and 1.1% of the measured variance in serum FSH, Inhibin B, testosterone and total testes volume, respectively. In contrast to the young male cohort, neither *FSHR* -29G/A nor *FSHR* haplotypes appeared to systematically modulate the reproductive physiology of oligozoospermic idiopathic infertile patients (n = 641, Estonians; aged 31.5±6.0 years). In summary, this is the first study showing the significant effect of *FSHR* -29G/A on male serum FSH level. To account for the genetic effect of known common polymorphisms modulating FSH-action, we suggest haplotype-based analysis of *FSHR* SNPs (*FSHR* -29G/A, c.2039 A/G) in combination with *FSHB* -211G/T testing.

## Introduction

Follicle-stimulating hormone (FSH) secreted by anterior pituitary together with other endocrine factors plays a central role in establishing and maintaining human fertility. Circulating FSH stimulates gametogenesis and steroidogenesis in gonads by binding into its receptor (FSHR). During male fetal, neonatal and pubertal periods, FSH stimulates proliferation of testicular Sertoli cells determining spermatogenic capacity of adult testes, and in adulthood it contributes to normal spermatogenesis and spermatogonial survival and sperm release [1], [2]. Inactivating mutations in the FSH β-subunit coding *FSHB* and the FSH receptor coding *FSHR* genes result in severely impaired spermatogenesis [3], [4]. In addition to loss-of-function variants, common polymorphisms in these genes have been shown to contribute to male reproductive physiology [5]. We have previously shown that the T-allele of the *FSHB* -211G/T promoter variant (rs10835638) was associated with significantly reduced serum FSH levels and total testes volume in the Baltic cohort of young men [6], [7], and these results were confirmed in Estonian, Italian and German infertile male patients [8]–[10]. Recent studies have also conclusively shown the association between the Ser680-allele of *FSHR* 2039A>G (p.Asn680Ser, rs6166) and higher serum FSH, lower total testes volume, Inhibin B and total testosterone levels [11], [12]. The *FSHR* c.2039A>G and the linked variant *FSHR* c.919A>G (p.Thr307Ala, rs6165) jointly determine the two FSHR isoforms [13].

**Figure 1:**
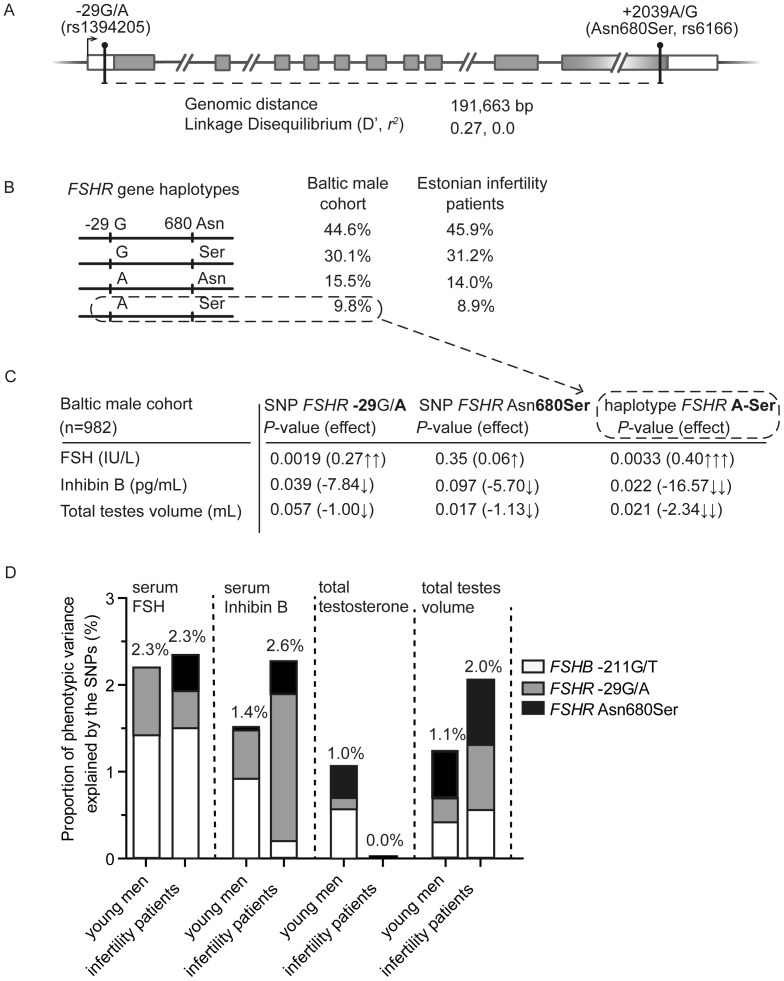
Genomic structure of the *FSHR* and effect of the FSH-action modulating genetic variants on reproductive parameters. **A**, Schematic representation of the structure of the *FSHR* gene drawn to an approximate scale. Exons are depicted as boxes, translated sequences are shaded in grey and transcription start-site is denoted with up-right arrow. Circle-headed bars indicate the location of *FSHR* SNPs -29G/A and +2039A/G (Asn680Ser); and their mutual genomic distance and linkage disequilibrium is provided. **B**, Distribution of the four *FSHR* haplotypes (G-Asn, G-Ser, A-Asn, A-Ser) formed from the *FSHR* -29G/A and +2039A/G (p.Asn680Ser) variants in the Baltic male cohort and Estonian oligozoospermic infertility patients (number of phased chromosomes, n = 1964 and n = 1282, respectively). **C**, Individual allelic effects of the *FSHR* -29 A-allele and *FSHR* 680Ser (c. +2039 G), and the cumulative haplotypic effect of the formed *FSHR* A-Ser gene variant on serum FSH, Inhibin B and total testes volume in the Baltic young men cohort. Results of the association testing are presented as *P*-values and effect sizes (regression coefficient, β) from linear regression analysis. Arrows indicate the strength and direction of the effects. **D**, Proportion of total phenotypic variance (%) of serum FSH, Inhibin B, total testosterone and total testes volume explained by the *FSHB* -211G/T (white bars), *FSHR* -29G/A (grey bars) and *FSHR* Asn680Ser (black bars) genetic variants in the Baltic young male cohort. Individual and cumulative effects of the SNPs were estimated by using the REML analysis implemented in GCTA software [33].

Another common polymorphism *FSHR* -29G/A (rs1394205) in the 5′-untranslated region of the gene (**Figure 1A**) has been reported to affect its transcriptional activity [14]. Although the discovery study in women had concluded that this SNP has little impact on FSHR expression and gonadal function [15], the follow-up clinical studies in female patients have showed that the expression level of the FSHR on human granulosa cells obtained from AA-genotype carriers was only 40% compared to the GG-genotype carriers [16] and fittingly, the amount of exogenous FSH required for ovulation induction was 1.8-fold higher in AA-homozygotes [16], [17]. The data on the effect of *FSHR* -29G/A in men is limited. Despite studies on several populations [18]-[22] and meta-analyses across individual reports [20], [23] have failed to identify significant contribution of the *FSHR* -29G/A on male infertility, there is missing data on the effect of this SNP on a wider range of male reproductive parameters. A pilot study has reported smaller testicular volume in Estonian men carrying the *FSHR* -29 A-allele [20].

We set forward to bring conclusive evidence to the effect of *FSHR* -29G/A in men in analysing a large study group (n = 1,623) comprising of Baltic young male cohort (n = 982) in comparison with Estonian oligozoospermic idiopathic infertile male patients (n = 641). There is emerging data on the importance of SNP-SNP and gene-gene interactions within relevant biological pathway(s) in determination of the studied phenotypic variation [24]. We performed haplotype-based association analyses combining the *FSHR* -29G/A genotype data reported in this study with the previously published dataset of the *FSHR* c.2039A>G (p.Asn680Ser) genotypes on the same samples [12]. In addition, the study groups were subjected to analysis of the joint contribution to normal phenotypic variance of the three main genetic variants reported to modulate the FSH action (*FSHB* -211G/T, *FSHR* -29G/A, *FSHR* c.2039A>G) [5]. To our knowledge, this is the first study showing the significant effect of *FSHR* -29G/A alone and in combination with *FSHR* c.2039A>G and *FSHB* -211G/T on male serum FSH level and downstream reproductive parameters.

## Materials and Methods

### Ethics statement

The study has been approved by the Ethics Committee of Human Research of the University Clinic of Tartu, Estonia (approval date 27.01.2003), the Ethics Committee of Riga Stradins University, Latvia (23.04.2003), and the Regional Ethics Committee of Kaunas, Lithuania (approval no. 13, 2003).

### The Baltic young male cohort

The Baltic male cohort was recruited between May 2003 and June 2004 among the participants in a prospective study Environment and Reproductive Health (EU 5th FP project QLRT-2001-02911) in parallel at three study centres (Tartu, Estonia; Riga, Latvia; Kaunas, Lithuania). The recruitment and phenotyping protocols at the participating centres were identical. Study participation was voluntary and written informed consent was obtained from all subjects. Details of the study group formation were described previously [25]. Men were recruited to the study at the Centre of Andrology, University Clinic of Tartu, Estonia (n = 578; all born and living in Estonia), at the Riga Family and Sexual Problems Centre, Latvia (n = 300; all born and living in Latvia), and at the specialized laboratory of the Institute of Endocrinology, Kaunas University of Medicine (n = 326; all born and living in Lithuania). Previously, either the sub-cohort of Estonian men or the full cohort of Baltic young men has been investigated for two SNPs modulating FSH-action, *FSHB* -211G/T [6], [7] and *FSHR* c.2039A>G [12]. In genetic association studies we have excluded cohort participants with clinical factors leading strongly deviated reproductive physiology (lack of sperm in ejaculate, i.e. azoospermia, n = 2; cryptorchidism, n = 13; abuse of anabolic steroids, n = 1; orchitis with unilateral testis damage, n = 1) or incomplete clinical data (n = 15). In addition, for the current study the DNA samples of a subset (n = 190) of the full Baltic male cohort were not available. The final number of analysed Baltic young male cohort participants for *FSHR* -29G/A (rs1394205) was 982 (**Table 1**).

**Table 1:** General characteristics of the study groups.

Parameter	Baltic male cohort (n = 982)		Estonian oligozoospermic patients (n = 641)	
General characteristics	mean±SD	median(5–95th percentile)	mean±SD	median(5–95th percentile)
Age (years)	20.2±2.0	19.8(17.4–24.2)	31.5±6.0	30.9(23.4–42.0)
BMI (kg/m^2^)	22.3±2.5	22.1(18.8–27.0)	26.6±4.4^a^	25.9(20.7–34.9)
Abstinence period (hours)	107.7±63.2	86.0(48.0–231.4)	92.3±51.0	72.0(48.0–168.0)
Total testes volume (mL)	49.2±10.3	50.0(33.0–70.0)	40.3±10.3	40.0(24.0–56.0)
Sperm concentration (10^6^/mL)	81.7±74.4	63.3(9.2–214.3)	7.8±5.9	7.0(0.1–18.0)
*FSHR* -29G/A (rs1394205)^b^				
Allele frequencies				
G	74.8 (1466)		77.1 (988)	
A	25.4 (498)		22.9 (294)	
?^2^-test^c^			*P* = 0.12	
Genotype frequencies				
G/G	56.2 (552)^d^		59.3 (380)^e^	
G/A	36.9 (362)		35.6 (228)	
A/A	6.9 (68)		5.1 (33)	
?^2^-test^c^			*P* = 0.12	

a Data for BMI available for 324 patients of the oligozoospermic study group.

b Data presented as percentage with number of allele/genotype carriers in brackets.

c *P*-value from χ^2^-test for differences in *FSHR* -29G/A allele and genotype distribution between Estonian oligozoospermic infertile patients and Baltic male cohort.

d Hardy-Weinberg Equilibrium test *P*-value of  = 0.4.

e Hardy-Weinberg Equilibrium test *P*-value of  = 1.0.

### Estonian idiopathic infertility patients

The study group of oligozoospermic Estonian men with idiopathic infertility (n = 750) was recruited at the Andrology Centre, Tartu University Clinics between June 2003 and August 2008 and consisted of male partners of couples failing to conceive a child for a period of ≥12 months. Oligozoospermia was diagnosed according to the World Health Organization (WHO) criteria valid at the time of recruitment (sperm concentration <20 mln/mL [26]). Phenotyping protocol was identical with that in Baltic young male cohort [25]; the details of the formation of the study group are described elsewhere [27]. In brief, all study participants were of white European ancestry, born and living in Estonia. All men with causal factors for male factor infertility (obstruction, cryptorchidism, chromosomal abnormalities, Y chromosome deletions, hypogonadotrophic hypogonadism, testicular diseases, sexual dysfunctions, androgen abuse, severe traumas and operation in genital area, chemo- and radiotherapy) were excluded from the analyses resulting in a study group consisting of 688 participants. For the current project, also patients with azoospermia, i.e. lack of sperms in ejaculate, n = 47) were additionally excluded from the genetic analysis. The final number of analysed subjects successfully genotyped for *FSHR* -29G/A (rs1394205) was 641. Among the included infertility patients (sperm concentration <20 mln/mL), 408 subjects also fulfilled the latest WHO 2010 criteria for oligozoospermia (sperm count below 39×10^6^/ejaculate [28]).

### Hormone assays

For all participants of the study, venous blood was obtained from the cubital vein in the morning and serum was separated immediately. Serum sampling period for the Baltic cohort was from 08.00 to 13.00 (median 11.00), and for the Estonian infertility patients from 08.00 to 11.00 (median 9.30), respectively.

For the Baltic cohort, serum levels of FSH, LH and total testosterone were determined using time-resolved immunofluorometric assays (Delfia, Wallac, Turku, Finland), estradiol by radioimmunoassay (Pantex, Santa Monica, CA, USA) and Inhibin B by a specific two-sided enzyme immunometric assay (Serotec, Oxford, UK) at the Department of Growth and Reproduction in Copenhagen, Rigshospitalet, Denmark in the framework of the Environment and Reproductive Health (EU 5th FP project QLRT-2001-02911). The intra- and inter-assay coefficients of variation (CV) for measurement of both FSH and LH were 3 and 4.5%, for total testosterone <8% and <5%, for estradiol 7.5% and 13%, and for Inhibin B 15% and 18%, respectively.

For the Estonian idiopathic infertility patients, the FSH, LH, total testosterone and estradiol levels of blood serum were measured using the Immulite automated chemiluminescence immunoassay analyser (Immulite; Diagnostic Products Corp., Los Angeles, CA) according to manufacturer's instructions, at the United Laboratories, University of Tartu Clinics. At the same laboratory, Inhibin B was determined in duplicate using a specific enzyme immunometric assay (Diagnostic Systems Laboratories, Inc., Webster, TX). The intra- and inter-assay CV were 4.2 and 8% for FSH; 4.0 and 7.1% for LH; 6.3 and 9.4% for testosterone; 7.5% and 13% for estradiol; 15% and 18% for Inhibin B.

Testing of genetic association between the *FSHR* -29G/A (rs1394295) SNP and serum hormone levels was carried out separately for the Baltic young men cohort and Estonian idiopathic infertility patients.

### Semen analysis and physical examination

Semen samples were obtained by masturbation and all semen values were determined in accordance with the World Health Organization (WHO) criteria valid at the time of recruitment [26]. In brief, after ejaculation, the semen was incubated at 37°C for 30–40 min for liquefaction. Semen volume was estimated by weighing the collection tube with the semen sample and subsequently subtracting the predetermined weight of the empty tube assuming 1 g = 1 mL. For assessment of the sperm concentration, the samples were diluted in a solution of 0.6 mol/L NaHCO3 and 0.4% (v/v) formaldehyde in distilled water. The sperm concentration was assessed using the improved Neubauer haemocytometers.

Patients were examined by clinical investigators who had passed special clinical training. Physical examination for the assessment of genital pathology and testicular size was performed with the man in standing position. If necessary, pathologies were clarified further with the men in supine position. The orchidometer (made of birch wood, Pharmacia & Upjohn, Denmark) was used for the assessment of testicular size. The total testes volume is the sum of right and left testicles.

### Genotyping procedure and data

Genomic DNA was extracted from peripheral blood using a modified version of the salting-out method [29]. The *FSHR* -29G/A (rs1394205) polymorphism was genotyped by PCR and allelic discrimination assay on the ABI PRISM 7900HT detection system (Applied Biosystems, Foster City, CA, USA). Each PCR reaction (5 μl) contained 20 ng genomic DNA, 1 μl 5x HOT FIREPol Probe qPCR Mix Plus (ROX) (Solis BioDyne, Tartu, Estonia), 0.25 μl predesigned TaqMan SNP Genotyping Assay (ID: C_426553_10; Applied Biosystems, Foster City, CA, USA) and 3.75 μl ddH_2_0. Default thermal cycling conditions were applied: 15 min at 95°C followed by 40 cycles of 15 sec at 95°C, 1 min at 60°C, and 2 min at 50°C. Results were analysed using the allelic discrimination endpoint analysis mode of the Sequence Detection software package, Version 2.4 (SDS 2.4). Quality control of genotyping was guaranteed by inclusion of positive control DNAs (previously sequenced) representing the three *FSHR* -29G/A genotypes (GG, GA, AA) into each genotyping plate.

Previously published genotyping data for *FSHR* Asn680Ser (c.2139 A/G, rs6166; **Table S1**; [12]) and the *FSHB* -211G/T (rs10835638; **Table S2**; [7]) were integrated into the *FSHR* haplotype (-29G/A–c.2139 A/G) analysis and in estimating the cumulative effect of all the three FSH-action modulating SNPs on phenotypic variance of the studied parameters.

### Data analysis

Mean, standard deviation, median, and 5–95^th^ percentiles were calculated for general characteristics (age, BMI, ejaculation abstinence period) and main outcome variables (hormonal and seminal variables, total testes volume) using PASW software Grad Pack 18.0 (SPSS Inc., Chicago, IL, USA). Genepop software (Version 4.0.10) was used test concordance of the genotyping data with Hardy-Weinberg equilibrium, and to test population differentiation (χ2-test) between the two study groups [30]. The testing conditions were: dememorization = 10000, batches = 1000, iterations = 10000.

Genetic associations between the studied individual SNPs and male reproductive parameters were tested using multiple linear regression implemented in PLINK, version 1.07 (http://pngu.mgh.harvard.edu/purcell/plink/) [31]. The natural log-transformation was used to obtain an approximate normal distribution of values for all studied quantitative parameters except total testes volume. Single SNP association tests were performed under additive genetic model. In the Baltic male cohort, regression testing was performed with the adjustment for age, BMI, smoking status, and recruitment centre. Hormone measurements were additionally corrected for blood sampling hour, and semen parameters were corrected for ejaculation abstinence period according to the analysis settings described previously [7]. In case of Estonian idiopathic infertile patients, linear regression was performed with adjustment for age; semen parameters were additionally corrected for abstinence period. The Bonferroni threshold for correction for multiple testing in linear regression analysis was estimated 0.05/16 = 3.13×10^−3^, taking into account the number of independent measurements (eight) and tested study samples (two).

The amount of LD between the *FSHR* -29G/A (rs1394205) and Asn680Ser (rs6166) was calculated using *r^2^* and D′ statistics implemented in Haploview [32]. The *FSHR* gene haplotypes comprised of alternative alleles of the *FSHR* -29G/A and c.2039 A/G (p.Asn680Ser) and subsequent haplotype frequencies were inferred from unphased genotype data using expectation-maximization (EM) algorithm implemented in PLINK, ver. 1.07 [31]. Tests for associations of the inferred *FSHR* -29G/A – c.2039 A/G haplotypes and male reproductive traits were conducted using haplotype-based linear regression analysis implemented in PLINK by ‘—hap-linear’ option based on a sliding-window approach with two included SNPs. Haplotype-based analyses were adjusted for appropriate cofactors analogously to the individual SNP association tests. Correction for multiple testing was performed using Max (T) permutation procedure (permutations = 10,000). Haplotype omnibus tests were performed testing for an overall effect of the haplotypes on the studied parameter.

Non-parametric Mann-Whitney two-tailed *U*-test implemented in PASW software Grad Pack 18.0 (SPSS Inc., Chicago, IL, USA) was used in Baltic male cohort to assess statistical differences in serum FSH level and total testes volume between the carriers (GA+AA, n = 430) and non-carriers (GG homozygotes, n = 552) and of the *FSHR* -29 A-allele. Mann-Whitney *U*-test compares the medians and the distribution of values. The Bonferroni threshold for correction for multiple testing in Mann-Whitney *U*-test was estimated 0.05/8 = 6.25×10^−3^, taking into account the number of independent measurements (eight).

The proportion of total phenotypic variance of studied hormonal and testicular parameters explained by the *FSHB* -211G/T, *FSHR* -29G/A and *FSHR* Asn680Ser SNPs was estimated using the REML (restricted maximum likelihood) analysis implemented in Genome-wide Complex Trait Analysis (GCTA) software (http://www.complextraitgenomics.com/software/gcta/index.html) [33]. Briefly, Genetic relationship matrixes (GRMs) were calculated for each study sample to determine the genetic relationship between pairs of individuals. GRMs served as input into a restricted maximum likelihood (REML) analysis to produce estimates of the proportion of phenotypic variance explained by the studied SNPs (V_G_/V_P_). REML analyses were adjusted for appropriate cofactors analogously to the individual SNP association tests.

## Results

### *FSHR* -29 A-allele is not enriched among male infertility patients

*FSHR* -29G/A SNP (rs1394205) was genotyped in the Baltic male cohort (n = 982; age 20.2±2.0 years; sperm concentration 81.7±74.4 mln/mL) and in Estonian oligozoospermic patients diagnosed with idiopathic infertility (n = 641; age 31.5±6.0 years; sperm concentration 7.8±5.9 mln/mL; **Table 1**). The estimated allele and genotype frequencies of the *FSHR* -29G/A did not differ between the two study groups (minor allele frequency, MAF = 25.4% in Baltic cohort *vrs*. 22.9% in Estonian oligozoospermic patients; Fisher's exact test, *P* = 0.12; **Table 1**).

### *FSHR* -29G/A is a novel identified genetic determinant of serum FSH

Among the Baltic young men, linear regression analysis resulted in significant association of the A-allele of the *FSHR* -29G/A SNP with higher serum FSH (additive model; *P* = 0.0019, A-allele effect 0.27 IU/L, resistant to Bonferroni correction; **Table 2**). This was supported by statistical analysis comparing the two subgroups of patients stratified based on their carrier status of the *FSHB* -29 A-allele. The group of consisting of AA-homozygotes and GA-heterozygotes (n = 430) compared to GG-homozygotes (n = 552) had significantly higher serum FSH (median 2.9 vrs. 2.6 IU/L, Mann-Whitney *U*-test *P* = 0.004, resistant to Bonferroni correction; **Table 3**). Additionally, the A-allele of the *FSHR* -29G/A showed a non-significant trend (*P*<0.06) for association with increased serum LH (linear regression: effect 0.19 IU/L) and total testosterone (effect 0.93 nmol/L) levels, but lower Inhibin B (effect −7.84 pg/mL) and total testes volume (effect −1.00 mL) (**Table 2**). Serum estradiol and semen parameters (semen volume, sperm concentration and count) were not associated with the *FSHR* -29G/A variant.

**Table 2:** Marker-trait association analysis and clinical parameters of the two study samples stratified into subgroups based on the *FSHR* -29G/A (rs1394205) genotypes of the participants.

Parameter		Baltic male cohort^a^				Estonian oligozoospermic men^a^			
	Group	mean±SD	median(5–95%)	*P*-value	beta(SE)^b^	mean±SD	median(5–95%)	*P*-value	beta(SE)^b^
FSH (IU/L)	G/G	3.0±1.6	2.6(1.1–5.9)	0.0019*	0.27(0.07)	7.7±6.6	5.6(1.9–21.6)	0.098	−0.41(0.26)
	G/A	3.2±1.8	2.9(1.2–6.6)			6.9±5.3	5.5(1.9–17.5)		
	A/A	3.4±1.7	3.0(1.4–7.0)			6.3±4.5	4.7(1.2–17.8)		
LH (IU/L)	G/G	3.9±1.7	3.7(1.8–6.9)	0.021	0.19(0.08)	4.5±2.2	4.1(1.6–8.5)	0.50	−0.08(0.12)
	G/A	4.2±1.6	4.0(1.9–7.1)			4.2±1.8	3.9(1.8–7.5)		
	A/A	4.1±1.5	3.7(2.1–7.0)			4.5±1.9	4.0(1.7–8.3)		
Inhibin B (pg/mL)	G/G	234.6±83.1	225.0(115.0–391.6)	0.039	−7.84 (3.90)	87.3±54.9	78.2(17.5–179.4)	0.093	10.60(6.12)
	G/A	224.6±71.9	219.0(110.2–351.9)			100.9±58.6	92.0(11.5–197.0)		
	A/A	217.3±72.1	198.0(109.7–363.2)			117.5±106.7	78.0(15.6–374.5)		
Total testosterone (nmol/L)	G/G	27.1±9.5	25.9(14.5–44.3)	0.042	0.93(0.45)	18.6±5.9	18.0(10.5–29.0)	0.40	−0.35(0.42)
	G/A	27.8±8.4	26.9(14.6–42.9)			18.6±7.3	17.9(8.8–31.9)		
	A/A	28.6±9.9	27.5(16.3–51.1)			18.2±5.3	17.7(10.1–30.5)		
Estradiol (pmol/L)	G/G	92.9±25.8	89.0(58.0–141.0)	0.26	1.35(1.20)	99.2±36.1	87.0(73.0–155.8)	0.55	1.21(2.01)
	G/A	95.0±23.9	92.0(59.3–142.7)			101.8±40.2	88.1(73.0–171.3)		
	A/A	94.6±26.3	90.0(60.0–135.3)			100.0±31.9	90.2(73.0–170.0)		
Total testes volume (mL)	G/G	49.5±10.9	50.0(32.0–70.0)	0.057	−1.00(0.53)	39.4±9.6	40.0(22.0–55.0)	0.017	1.67(0.69)
	G/A	49.2±9.3	50.0(36.0–65.0)			41.5±11.4	41.0(25.0–59.3)		
	A/A	46.2±10.4	47.0(27.2–62.2)			41.6±10.1	41.0(24.2–62.4)		
Semen volume (mL)	G/G	3.5±1.6	3.4(1.3–6.4)	0.55	−0.05(0.08)	4.2±1.7	3.9(1.7–7.7)	0.53	0.07(0.12)
	G/A	3.6±1.7	3.3(1.3–6.6)			4.2±1.8	4.0(1.7–7.7)		
	A/A	3.4±1.7	3.0(1.4–6.5)			4.8±2.3	4.9(1.6–9.7)		
Sperm concentration (10^6^/mL)	G/G	85.5±82.2	64.3(9.2–225.4)	0.28	−2.94(2.85)	7.8±6.0	7.0(0.1–18.0)	0.37	0.43(0.47)
	G/A	77.4±65.4	63.0(8.2–202.0)			7.5±5.7	7.0(0.1–18.0)		
	A/A	72.5±48.0	62.6(7.1–148.9)			10.4±6.5	10.4(0.1–19.0)		
Total sperm count (10^6^/ejaculate)	G/G	288.5±298.3	219.5(19.1–780.8)	0.21	−11.79(9.91)	33.1±31.2	24.5(0.4–95.0)	0.30	1.99(1.91)
	G/A	264.0±252.4	198.3(19.1–719.0)			32.7±29.6	23.0(0.3–89.4)		
	A/A	235.3±184.5	195.9(18.1–662.0)			48.3±37.6	45.6(0.3–126.3)		

a Baltic young men cohort, n = 982, A-allele frequency 25.4%, HWE test *P* = 0.40; Estonian oligozoospermic men, n = 641, A-allele frequency 22.9%, HWE test *P* = 1.0.

b *FSHR* -29 A-allele effect is shown as the estimated linear regression (additive model) statistic beta (β), standard error of the regression (SE) is shown in brackets. Asterisk (*) indicates a significant association, *P*<0.05 after Bonferroni correction for multiple testing.

**Table 3:** Test results for the difference in estimated median values and distributions of the study parameters in the Baltic male cohort subgroups stratified based on the carrier status of the A-allele of *FSHR* -29G/A.

Parameter^a^	*FSHR* -29 GG-carriers (n = 552)	*FSHR* -29 A-carriers (GA+AA, n = 430)	*P*-value^b^
FSH (IU/L)	2.6(1.1–5.9)	2.9(1.3–6.6)	0.004*
LH (IU/L)	3.7(1.8–6.9)	3.9(2.0–7.1)	0.016
Inhibin B (pg/mL)	225.0(115.0–391.6)	218.0(110.6–352.5)	0.091
Total testosterone (nmol/L)	25.9(14.5–44.3)	27.0(14.9–45.0)	0.049
Estradiol (pmol/L)	89.0(58.0–141.0)	91.5(60.0–141.0)	0.087
Total testes volume (mL)	50.0(32.0–70.0)	50.0(34.0–64.3)	0.435
Semen volume (mL)	3.4(1.3–6.4)	3.2(1.3–6.5)	0.781
Sperm concentration (10^6^/mL)	64.3(9.2–225.4)	62.8(8.5–201.6)	0.235
Total sperm count (10^6^/ejaculate)	219.5(19.1–780.8)	197.1(18.6–691.9)	0.112

a Data presented as median(5–95^th^ percentile).

b Non-parametric Mann-Whitney two-tailed *U*-test implemented in PASW software Grad Pack 18.0; asterisk (*) marks *P*-values resistant to Bonferroni correction for multiple testing.

In contrast to healthy young male cohort, no genetic associations between *FSHR* -29G/A and tested reproductive parameters reached Bonferroni-corrected statistical significance level irrespective whether oligozoospermia was diagnosed according WHO 1999 criteria used at the recruitment (sperm concentration below 20×10^6^/mL [26], **Table 2**) or based on the revised WHO 2010 edition (sperm count below 39×10^6^/ejaculate [28], **Table S3**). Interestingly, among infertility patients, the *FSHR* -29 A-allele showed a trend for association with higher total testes volume (oligozoospermia based on WHO 1999, nominal *P* = 0.017, **Table 2**; WHO 2010, *P* = 0.043, **Table S3**).

### Gene haplotypes formed from *FSHR* -29G/A and c.2039 A/G (p.Asn680Ser) exhibit enhanced effect on serum hormones and testes volume compared to individual SNPs

We aimed at the haplotype-based association analyses combining the *FSHR* -29G/A genotype data reported in the current study with our previously published the *FSHR* c.2039A>G (p.Asn680Ser) genotype data of the same samples [12] (**Table S1**). The common polymorphisms *FSHR* -29G/A (in 5′UTR) and *FSHR* c.2039 A/G (p.Asn680Ser, exon 10) locate >190 kb apart and showed linkage disequilibrium (LD; D′ = 0.027; *r^2^* = 0.0) neither in the Baltic male cohort nor in Estonian oligozoospermic patient study group (**Figure 1A**). The four *FSHR* common variants arise from alternative allelic combinations of the *FSHR* -29G/A and the *FSHR* c.2039 A/G SNPs: -29A/2039A (A-Asn), -29A/2039G (A-Ser), -29G/2039A (G-Asn), and -29G/2039G (G-Ser). The estimated frequency distribution of the four haplotypes did not differ between the young men and oligozoospermic male patients (**Table 4** **,** **Figure 1B**). The most prevalent haplotype was -29G/2039A (G-Asn) with 44.6% and 45.9% carrier frequency in the two study groups, respectively.

**Table 4:** Effect of the inferred *FSHR* gene haplotypes on tested male hormonal and testicular parameter distribution.

		Baltic male cohort (n = 982)					Estonian oligozoospermic patients (n = 641)				
		Effect of individual *FSHR* haplotypes^a^				Overall effect of the haplotypes^b^	Effect of individual *FSHR* haplotypes^a^				Overall effect of the haplotypes^b^
Parameter		G-Asn (44.6%)	G-Ser (30.1%)	A-Asn (15.5%)	A-Ser (9.8%)		G-Asn (45.9%)	G-Ser (31.2%)	A-Asn (14.0%)	A-Ser (8.9%)	
FSH (IU/L)	*P*-value^c^ (beta^d^)	0.16 (-0.13)	0.69 (−0.07)	0.43 (0.14)	**0.0033 (0.40)**	**0.007**	0.75 (−0.21)	**0.037 (0.66)**	0.30 (−0.55)	0.86 (−0.01)	**0.045**
LH (IU/L)	*P*-value (beta)	0.80 (−0.06)	0.44 (0.20)	0.22 (0.19)	0.35 (0.20)	0.14	0.81 (−0.09)	0.36 (0.18)	1.00 (−0.02)	0.72 (−0.20)	0.28
Inhibin B (pg/mL)^e^	*P*-value (beta)	0.13 (7.69)	1.00 (−0.48)	0.96 (−2.36)	**0.022 (−16.57)**	**0.043**	0.79 (4.28)	**0.048 (−12.32)**	0.12 (20.86)	0.96 (4.50)	0.068
Total testosterone (nmol/L)	*P*-value (beta)	0.99 (0.14)	0.06 (0.33)	0.067 (1.47)	0.96 (0.33)	0.057	0.63 (0.43)	0.97 (−0.16)	1.00 (−0.09)	0.60 (−0.78)	0.62
Estradiol (pmol/L)	*P*-value (beta)	1.00 (0.40)	0.37 (0.23)	0.53 (2.28)	1.00 (0.23)	0.41	0.99 (0.54)	0.77 (−1.64)	0.81 (2.18)	1.00 (−0.02)	0.81
Total testes volume (mL)	*P*-value (beta)	**0.015** **(1.34)**	0.73 (−0.50)	0.99 (0.11)	**0.021 (−2.34)**	**0.018**	1.00 (0.09)	0.10 (−1.47)	**0.014 (3.02)**	1.00 (−0.04)	**0.023**
Semen volume (mL)	*P*-value (beta)	1.00 (−0.01)	0.82 (−0.15)	1.00 (0.02)	0.58 (−0.15)	0.51	0.64 (0.12)	0.24 (−0.19)	0.78 (0.14)	1.00 (0.00)	0.27
Sperm concentration (10^6^/mL)	*P*-value (beta)	0.18 (2.60)	1.00 (0.19)	0.20 (−6.55)	0.95 (2.28)	0.19	0.99 (−0.08)	0.82 (−0.29)	0.93 (0.30)	0.64 (0.73)	0.54
Total sperm count (10^6^/ejaculate)	*P*-value (beta)	0.30 (8.03)	0.98 (−1.72)	0.32 (−19.60)	1.00 (−1.72)	0.42	0.99 (0.38)	0.38 (−2.26)	0.79 (2.05)	0.67 (3.09)	0.26

a *FSHR* gene haplotypes were inferred using genotype data on *FSHR* -29G/A (rs1394205; Table 2) and *FSHR* 2039 A/G (rs6166, Asn680Ser; Table S1; (16). Haplotype G-Asn is the combination of G- and Asn-alleles at the *FSHR* positions -29G/A and Asn680Ser, respectively, etc.

b *P*-value from omnibus test estimating the overall effect of *FSHR* haplotypes on tested parameter distribution [30] (http://pngu.mgh.harvard.edu/~purcell/plink/).

c Corrected empirical *P*-value from haplotype-based association test for individual haplotypes after correction for multiple haplotypes using max(T) permutation procedure (number of permutations, n = 10,000).

d Effect of individual haplotypes is shown as the estimated linear regression (additive model) coefficient, β.

e Among Estonian oligozoospermic infertile patients, Inhibin B values were available for 264 individuals.

We addressed the overall effect of the *FSHR* -29G/A – c.2039 A/G inferred haplotypes (allelic combinations of these two polymorphisms) on male hormonal and testicular parameters, as well as analysed the individual effect of the four haplotypes (**Table 4**). Overall, in both study groups the carrier status of *FSHR* haplotypes exhibited a significant effect on serum FSH (haplotype omnibus test: Baltic cohort, *P* = 0.007; Estonian oligozoospermic infertile patients, *P* = 0.045), and total testes volume (*P*<0.03). In young men, the *FSHR* haplotypes also affected significantly serum Inhibin B levels (*P* = 0.043). Among individual haplotypes, the least common *FSHR* variant (A-Ser; frequency 9.8%) in young men was associated with significantly higher serum FSH (*P* = 0.0033, haplotype effect size 0.40 IU/L), but lower Inhibin B (*P* = 0.022, effect −16.57 pg/mL) and total testes volume (*P* = 0.021, effect −2.34 mL). The joint effect of the two genetic variants on each parameter was cumulative, e.g. *FSHR* -29 A allele effect on FSH is 0.27 IU/L, *FSHR* Ser-variant effect 0.06 IU/L and their haplotype effect 0.4 IU/L (**Figure 1C**). In case of total testes volume, the effect of minor alleles of these SNPs and their formed haplotype is additively negative: −1.00, −1.13 and −2.34 mL, respectively (**Figure 1C**). In contrast, the most prevalent *FSHR* G-Asn gene variant (44.6%) was positively correlated with larger total testes volume (*P* = 0.015, effect 1.34 mL).

**Figure 2:**
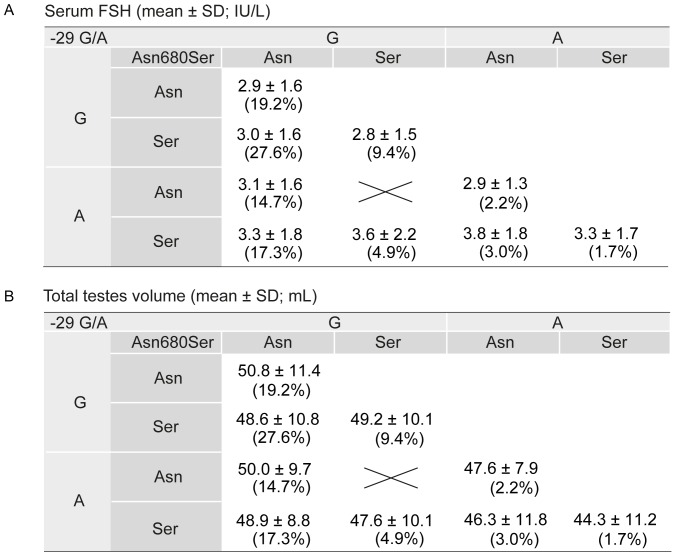
Effect of the carrier status of *FSHR* -29G/A and Asn680Ser genotype combinations on reproductive parameters. Effect of the *FSHR* -29G/A (rs1394205) and *FSHR* Asn680Ser (c.2039A>G, rs6166) genotype combinations on (**A**) serum FSH level (IU/L; mean ± SD) and (**B**) total testes volume (mL; mean ± SD) among the Baltic male cohort sample (n = 982). The -29G/A and Asn680Ser variants form nine possible *FSHR* genotype combinations: GG-AsnSer (n = 271), GG-AsnAsn (n = 189), AG-AsnSer (n = 170), AG-AsnAsn (n = 144), GG-SerSer (n = 92), AG-SerSer (n = 48), AA-AsnSer (n = 29), AA-AsnAsn (n = 22), AA-SerSer (n = 17). The prevalence of each genotype combination (%) is shown in brackets.

The results of inferred haplotype analysis are in line with the data of young men stratified based on the carrier status of nine alternative genotype combinations of the *FSHR* -29G/A and Asn680Ser polymorphisms (**Figure 2**). The carriers of both, *FSHR* -29A and 680Ser-variants exhibited significantly higher level of serum FSH (Mann-Whitney *U*-test *P* = 2.5×10^−3^; **Figure 2A**) and a trend for lower total testes volume (*P*<0.1; **Figure 2B**) compared to the rest of the study subjects. The homozygotes for the minor alleles of both *FSHR* variants (AA-SerSer) were measured 13% lower total testes volume compared to the subjects with the wild-type homozygote genotype GG-AsnAsn (mean 44.3 *vrs* 50.8 mL; *P*<0.1).

Among the Estonian oligozoospermic infertility patients, none of the four *FSHR* haplotypes appeared to systematically modulate their reproductive physiology (**Table 4**). We observed single genetic associations for the two haplotypes with intermediate prevalence (G-Ser, A-Asn), but these cannot be equivocally explained by the deviated reproductive physiology in infertile men, and the significant *P*-values (0.01<*P*<0.05) may have been detected by chance only. Infertile patients carrying *FSHR* G-Ser haplotype (frequency 31.2%; high gene expression, but less sensitive receptor) had higher serum FSH and lower Inhibin B levels, whereas haplotype A-Asn (14.0%; low gene expression, but more sensitive receptor) was associated with larger total testes volume (**Table 4**).

### Phenotypic variance of reproductive parameters in young men explained by FSH-action modulating genetic variants

The contribution of the three FSH-action modulating genetic variants (*FSHB* -211G/T, *FSHR* -29G/A and c.2039A>G) to the measured population variance in serum reproductive hormone levels, total testicular volume and sperm concentration was estimated in the Baltic young male cohort using the REML analysis implemented in GCTA software [33]. Here, we took advantage of our previously published datasets on the *FSHR* c.2039A>G (p.Asn680Ser; **Table S1**; [12]) and the *FSHB* -211G/T (**Table S2**; [7]) genotype data of the same samples. Together, the three SNPs explained 2.3%, 1.4% and 1.0% of the phenotypic variance in circulating FSH, serum Inhibin B and total testosterone levels, respectively, with the *FSHB* -211G/T accounting for the largest proportion of the variance (serum FSH, 1.4%; serum Inhibin B, 0.9%; total testosterone, 0.6%) (**Figure 1D**). For the total testes volume, the *FSHB* and *FSHR* SNPs together accounted for 1.1% of the phenotypic variance. The *FSHB* and *FSHR* variants almost equally contributed to the variance in total testes volume (*FSHR* c.2039A>G, 0.5%; *FSHB* -211G/T, 0.4%; *FSHR* -29G/A, 0.3%). In agreement with the data on individual SNPs [7], [12], the estimated joint effect on sperm concentration is marginal (0.2%, data not shown).

## Discussion

FSH action in male and female reproductive physiology is modulated by genetic variants determining either serum FSH levels or the functionality of FSHR [5]. Previously, few small-scale studies have inconclusively investigated the effect of the *FSHR* -29G/A alone or in combination with other *FSHR* genetic variants on male quantitative reproductive parameters [18]–[22]. This the first study to show significant associations between the *FSHR* 29G/A genetic variant and reproductive hormone levels in men. For the Baltic young male cohort, we report statistically significant association of the *FSHR* -29 A-allele with higher serum FSH. The results fit with the published data on higher gene expression level for the *FSHR* -29 G-allele compared to A-allele [16], and suggests that in the latter case there might be shortage of FSHR molecules to bind the circulating FSH. The genetic effect of *FSHR* -29G/A was enhanced in haplotype-based association analysis, which highlighted the *FSHR* haplotype -29A/2039G (A-Ser; lower transcript level and less sensitive receptor) to modulate reproductive physiology. It was associated with statistically higher FSH (effect 0.40 IU/L), lower Inhibin B (effect −16.57 pg/mL) and smaller total testes volume (effect −2.34 mL). Consistently, the most prevalent haplotype, G-Asn, combining the effect of higher *FSHR* gene expression and increased FSH receptor ligand-sensitivity was significantly and cumulatively associated with larger testes volume (effect 1.34 mL). Thus, our study demonstrates the importance of taking into account the cumulative effect of both common genetic variants in the *FSHR* gene, and suggests the haplotype-based association analysis instead of single SNP testing.

To our knowledge, this is the first study aiming at estimating the genetic contribution to the normal phenotypic variance in circulating FSH level and total testes volume in general population. We showed that in healthy young men, the three analysed SNPs (*FSHR* -29G/A, c.2039 A/G; *FSHB* -211G/T) explain together 2.3%, 1.4%, 1.0 and 1.1% of the measured variance in serum FSH, Inhibin B, testosterone and total testes volume, respectively. Genome-wide association study (GWAS) of steroid hormone levels has reported 11 SNPs associated with estradiol to explain 6.5%, and 6 SNPs associated with testosterone to explain 4.4% of the variance in these hormone levels in postmenopausal women [34]. A GWAS on male serum testosterone levels reported three SNPs in the *SHBG* gene to explain 3.8% of its variance [35].

Consistent with the evidence from previous reports in other populations [18], [19], [21], [22] and from a recent meta-analysis combining seven studies (in total, 1,644 infertility cases and 1,748 controls) [23], the current study showed no statistical difference in allelic and genotypic distribution of *FSHR* -29G/A between the Baltic young men cohort (n = 982) and Estonian oligozoospermic idiopathic infertility patients (n = 641). Also the frequency distribution of the four *FSHR* haplotypes [formed from *FSHR* -29G/A and c.2039 A/G (p.Asn680Ser)] did not differ between the young men and oligozoospermic patients. Thereby, we conclude that there is a lack of the association between common genetic variants in the *FSHR* gene and substantial effect on male infertility risk. Notably, although the genetic composition in young male cohort and oligozoospermic patient group was similar, neither *FSHR* -29G/A alone nor *FSHR* variants formed from the combinations of -29G/A and Asn680Ser appeared to significantly modulate reproductive physiology among infertility patients (**Tables 2** **–** **4**). One possible scenario to explain the contrasting outcome of association testing is an important limitation that the hormonal parameters for the two study samples had been measured using different methods and laboratories. However, hormonal data of both samples were determined in accredited centres using standardized commercial assays and have been previously successfully applied in genetic association studies, providing mutually consistent results [6], [8]. A more probable scenario supports that in the group of highly selected patients with oligozoospermia and male factor infertility, the causes of their impaired reproductive function are heterogeneous and alternative biological pathways may be involved to maintain their fertility. Therefore one should be cautious when interpreting physiologically unsound effects of genetic variation on reproductive physiology in patients with infertility problems.

In summary, we conclusively showed the significant effect of the *FSHR* -29 A-allele on male serum FSH level. Effect on FSH and its downstream hormonal and testicular parameters were further cumulatively modulated by the carrier-status of the *FSHR* c.2039 G-allele (p.680Ser). Notably, our study highlights the importance in genetic studies of reproductive parameters to utilize population-based individuals with normal and undisturbed reproductive physiology. We estimated that the three FSH-action modulating genetic variants (*FSHR* -29G/A, c.2039 A/G; *FSHB* -211G/T) account for the substantial proportion of the total normal phenotypic variance in male reproductive parameters. Whether these polymorphisms may represent genetic risk factors to male reproductive disorders apart from fertility, but possibly affected by impaired FSH action (e.g. cryptorchidism, hypospadias, testicular dysgenesis syndrome and testicular cancer), has to be addressed in future studies.

## Supporting Information

Table S1 Marker-trait association analysis and clinical parameters of the two study groups stratified based on the *FSHR* Asn680Ser (rs6166) genotypes of participants.
(PDF)

Table S2 Marker-trait association analysis and clinical parameters of the Baltic male cohort sample stratified based on the *FSHB* -211G/T (rs10835638) genotypes of the participants.
(PDF)

Table S3 Marker-trait association analysis and clinical parameters of the subgroup of Estonian oligozoospermic idiopathic infertility study sample defined according to the World Health Organization 2010 criteria (sperm count below 39x10^6^/ejaculate; n = 408). Parameter data is provided for the subgroups of patients stratified based on their *FSHR* -29G/A, *FSHR* Asn680Ser and *FSHB* -211G/T genotypes.
(PDF)
