# Supplementary material for: Reproductive Physiology in Young Men Is Cumulatively Affected by FSH-Action Modulating Genetic Variants: FSHR -29G/A and c.2039 A/G, FSHB -211G/T
Source: PLoS One. 2014 Apr 9;9(4):e94244. doi: 10.1371/journal.pone.0094244 (PMC3981791; doi:10.1371/journal.pone.0094244)
Supplement: Table S1 — Marker-trait association analysis and clinical parameters of the two study groups stratified based on the FSHR Asn680Ser (rs6166) genotypes of participants. (PDF) [file pone.0094244.s001.pdf]

Supplementary Table S1. Marker-trait association analysis and clinical parameters of the two study groups stratified based on the *FSHR* Asn680Ser (rs6166) genotypes of participants.

Parameter[c]	FSHR Asn680Ser	Baltic male cohort[a] mean ± SD median (5-95)	Baltic male cohort[a] *P*-value beta (SE)[d]	Estonian oligozoospermic men[b] mean ± SD median (5-95)	Estonian oligozoospermic men[b] *P*-value beta (SE)[d]
FSH (IU/L)	Asn/Asn	3.0 ± 1.6		7.3 ± 6.1	
		2.6 (1.1 – 5.9)		5.5 (1.9 – 19.9)	
	Asn/Ser	3.2 ± 1.7	0.35	6.7 ± 5.4	0.056
		2.8 (1.1 – 6.5)	0.06 (0.06)	5.1 (1.7 – 15.7)	0.43 (0.22)
	Ser/Ser	3.1 ± 1.8		9.3 ± 7.4	
		2.7 (1.3 – 6.3)		6.8 (2.3 – 24.9)	
LH (IU/L)	Asn/Asn	4.1 ± 1.6		4.4 ± 2.1	
		3.8 (1.8 – 6.8)		4.1 (1.7 – 8.3)	
	Asn/Ser	4.0 ± 1.6	0.69	4.2 ± 1.9	0.39
		3.9 (1.8 – 7.0)	-0.03 (0.07)	3.7 (1.6 – 7.6)	0.09 (0.11)
	Ser/Ser	4.0 ± 1.8		4.8 ± 2.4	
		3.8 (1.8 – 7.5)		4.3 (2.1 – 8.7)	
Inhibin B (pg/mL)	Asn/Asn	235.0 ± 75.4		93.6 ± 49.9	
		227.0 (121.8 – 381.8)		93.4 (10.4 – 182.7)	
	Asn/Ser	224.1 ± 77.2	0.097	98.4 ± 65.8	**0.047**
		214.0 (111.5 – 361.5)	-5.70 (3.51)	81.0 (24.3 – 227.0)	-9.12 (5.04)
	Ser/Ser	234.5 ± 88.3		74.9 ± 51.1	
		222.0 (107.0 – 399.8)		55.3 (10.0 – 175.6)	
Total testosterone (nmol/L)	Asn/Asn	28.5 ± 8.7		19.1 ± 6.6	
		27.8 (14.9 – 45.2)		18.5 (9.9 – 30.0)	
	Asn/Ser	26.8 ± 9.6	0.059	18.3 ± 6.3	0.33
		25.5 (14.3 – 44.1)	-0.76 (0.41)	17.6 (10.0 – 30.8)	-0.34 (0.36)
	Ser/Ser	27.0 ± 8.7		18.4 ± 6.2	
		25.5 (15.2 – 45.3)		17.7 (10.2 – 29.1)	
Estradiol (pmol/L)	Asn/Asn	95.9 ± 26.7		104.0 ± 45.0	
		92.0 (59.0 – 144.2)	0.20	87.0 (73.0 – 178.6)	0.42
	Asn/Ser	92.7 ± 25.0	-1.37 (1.08)	97.3 ± 32.4	-1.34 (1.70)
		89.0 (59.0 – 140.5)		85.2 (73.0 – 152.0)	

	Ser/Ser	92.4 ± 21.3<br>91.0 (55.9 – 132.5)		100.2 ± 31.9<br>90.6 (73.0 – 168.0)	
Total testes volume (mL)	Asn/Asn	50.3 ± 10.5<br>50.0 (33.0 – 70.0)		41.1 ± 11.8<br>41.0 (24.0 – 59.1)	
	Asn/Ser	48.6 ± 10.2<br>50.0 (32.3 – 65.0)	**0.017**<br>-1.13 (0.48)	40.4 ± 9.3<br>40.0 (24.0 – 56.0)	**0.016**<br>-1.41 (0.59)
	Ser/Ser	48.2 ± 10.3<br>48.0 (31.7 – 70.0)		38.1 ± 9.7<br>37.0 (22.2 – 54.0)	
Semen volume (mL)	Asn/Asn	3.4 ± 1.6<br>3.1 (1.3 – 6.4)		4.4 ± 1.9<br>4.0 (1.8 – 8.0)	
	Asn/Ser	3.6 ± 1.6<br>3.4 (1.3 – 6.5)	0.97<br>0.00 (0.07)	4.2 ± 1.8<br>4.0 (1.5 – 7.7)	0.12<br>-0.15 (0.10)
	Ser/Ser	3.5 ± 1.7<br>3.4 (1.0 – 6.5)		4.0 ± 1.6<br>3.8 (1.3 – 7.2)	
Sperm concentration (10^6/mL)	Asn/Asn	79.0 ± 65.0<br>63.0 (7.0 – 206.6)		7.7 ± 5.6<br>7.0 (0.1 – 17.0)	
	Asn/Ser	84.3 ± 82.8<br>62.6 (9.2 – 224.3)	0.73<br>0.86 (2.57)	7.8 ± 6.0<br>7.0 (0.1 – 18.0)	0.71<br>-0.14 (0.40)
	Ser/Ser	79.3 ± 68.0<br>63.3 (12.1 – 189.1)		8.1 ± 6.6<br>7.0 (0.1 – 18.9)	
Total sperm count (10^6)	Asn/Asn	262.4 ± 237.7<br>202.2 (18.2 – 700.0)		33.4 ± 29.1<br>26.4 (0.5 – 90.8)	
	Asn/Ser	287.0 ± 298.2<br>214.9 (20.2 – 780.0)	0.79<br>2.28 (8.92)	34.3 ± 32.3<br>24.3 (0.3 – 96.8)	0.44<br>-1.16 (1.63)
	Ser/Ser	272.2 ± 285.1<br>216.7 (23.7 – 686.4)		32.8 ± 32.3<br>23.1 (0.4 – 96.0)	

[a] Baltic young men cohort, n=982; Ser-allele frequency 39.9%, HWE test *P*=0.95
[b] Estonian oligozoospermic men, n=641; Ser-allele frequency 40.2%, HWE test *P*=0.93
[c] Data presented as mean ± SD and median (5-95th percentile)
[d] *FSHR* 680Ser-allele effect is shown as the estimated linear regression (additive model) statistic β, standard error of the regression (SE) is shown in brackets. Significant associations (*P*<0.05) are given in bold; asterisk (*) marks *P*-values resistant to Bonferroni correction for multiple testing;
