# Supplementary material for: Reproductive Physiology in Young Men Is Cumulatively Affected by FSH-Action Modulating Genetic Variants: FSHR -29G/A and c.2039 A/G, FSHB -211G/T
Source: PLoS One. 2014 Apr 9;9(4):e94244. doi: 10.1371/journal.pone.0094244 (PMC3981791; doi:10.1371/journal.pone.0094244)
Supplement: Table S2 — Marker-trait association analysis and clinical parameters of the Baltic male cohort sample stratified based on the FSHB -211G/T (rs10835638) genotypes of the participants. (PDF) [file pone.0094244.s002.pdf]

Supplementary Table S2. Marker-trait association analysis and clinical parameters of the Baltic male cohort sample stratified based on the *FSHB* -211G/T (rs10835638) genotypes of the participants.

Parameter[b]	*FSHB* -211 G/T	Baltic male cohort[a]<br>mean ± SD<br>median (5-95)	 *P*-value<br>beta (SE)[c]
FSH (IU/L)	G/G	3.2 ± 1.7<br>2.9 (1.3 – 6.5)	
	G/T	2.7 ± 1.5<br>2.3 (1.1 – 5.6)	**0.0000051***<br>-0.39 (0.09)
	T/T	2.4 ± 1.3<br>2.4 (0.9 – 6.1)	
LH (IU/L)	G/G	4.0 ± 1.6<br>3.8 (1.8 – 6.9)	
	G/T	4.1 ± 1.6<br>3.8 (1.7 – 7.1)	0.38<br>0.09 (0.11)
	T/T	5.2 ± 2.4<br>4.3 (2.5 – 11.9)	
Inhibin B (pg/mL)	G/G	234.4 ± 78.5<br>226.5 (118.2 – 380.7)	
	G/T	217.0 ± 76.5<br>208.0 (106.3 – 361.7)	**0.0011***<br>-16.21 (5.21)
	T/T	184.9 ± 84.3<br>160.5 (77.0 – 361.0)	
Total testosterone (nmol/L)	G/G	27.8 ± 9.1<br>26.9 (15.0 – 44.1)	
	G/T	26.4 ± 9.3<br>25.0 (13.7 – 45.6)	**0.0075**<br>-1.56 (0.61)
	T/T	24.9 ± 7.7<br>27.3 (11.7 – 34.6)	
Estradiol (pmol/L)	G/G	93.9 ± 25.1<br>90.0 (59.0 – 141.0)	
	G/T	92.8 ± 24.9<br>90.0 (57.3 – 138.7)	0.66<br>-0.71 (1.61)
	T/T	102.0 ± 32.8<br>105.0 (59.0 – 161.0)	
Total testes volume (mL)	G/G	49.4 ± 10.2<br>50.0 (33.0 – 70.0)	
	G/T	48.8 ± 10.5<br>50.0 (30.1 – 70.0)	**0.0085**<br>-1.84 (0.72)
	T/T	40.2 ± 12.9<br>40.5 (20.0 – 70.0)	
Semen volume (mL)	G/G	3.5 ± 1.6<br>3.3 (1.3 – 6.5)	0.33<br>0.11 (0.11)
	G/T	3.5 ± 1.6	

		3.4 (1.2 – 6.1)	
	T/T	4.2 ± 1.8	
		4.3 (1.3 – 6.8)	
Sperm concentration (10^6/mL)	G/G	83.2 ± 73.0	
		65.3 (8.2 – 215.0)	
	G/T	78.1 ± 80.3	0.39
		60.2 (10.2 – 200.4)	-3.10 (3.84)
	T/T	54.1 ± 41.4	
		51.7 (8.4 – 135.1)	
Total sperm count (10^6)	G/G	282.4 ± 287.5	
		216.1 (18.2 – 748.1)	
	G/T	255.2 ± 235.3	0.75 -4.06 (13.35)
		181.8 (25.7 – 765.3)	
	T/T	249.5 ± 223.0	
		185.1 (14.6 – 649.3)	

[a] Baltic young men cohort, n=982; T-allele frequency 12.9%, HWE test *P*=0.67

[b] Data presented as mean ± SD and median ($5-95^{th}$ percentile)

[c] *FSHB* -211 T-allele effect is shown as the estimated linear regression (additive model) statistic β, standard error of the regression (SE) is shown in brackets. Significant associations (*P*<0.05) are given in bold; asterisk (*) marks *P*-values resistant to Bonferroni correction for multiple testing
