# Supplementary material for: Reproductive Physiology in Young Men Is Cumulatively Affected by FSH-Action Modulating Genetic Variants: FSHR -29G/A and c.2039 A/G, FSHB -211G/T
Source: PLoS One. 2014 Apr 9;9(4):e94244. doi: 10.1371/journal.pone.0094244 (PMC3981791; doi:10.1371/journal.pone.0094244)
Supplement: Table S3 — Marker-trait association analysis and clinical parameters of the subgroup of Estonian oligozoospermic idiopathic infertility study sample defined according to the World Health Organization 2010 criteria (sperm count below 39x106/ejaculate; n = 408). Parameter data is provided for the subgroups of patients stratified based on their FSHR -29G/A, FSHR Asn680Ser and FSHB -211G/T genotypes. (PDF) [file pone.0094244.s003.pdf]

Supplementary Table S3. Marker-trait association analysis and clinical parameters of the subgroup of Estonian oligozoospermic idiopathic infertility study sample defined according to the World Health Organization 2010 criteria (sperm count below 39x10^{6}/ejaculate; n=408). Parameter data is provided for the subgroups of patients stratified based on their *FSHR* -29 (G>A), *FSHR* Asn680Ser and *FSHB* -211 G/T genotypes.

Parameter[a]	***FSHR* -29 G/A (rs1394205)**			***FSHR* Asn680Ser (rs6166)**			***FSHB* -211 G/T (rs10835638)**		
		**mean ± SD**<br>**median (5-95)**	***P*-value**<br>**beta (SE)[b]**		**mean ± SD**<br>**median (5-95)**	***P*-value**<br>**beta (SE)[b]**		**mean ± SD**<br>**median (5-95)**	***P*-value**<br>**beta (SE)[b]**
**FSH (IU/L)**	**G/G**	8.7 ± 7.6<br>6.2 (1.9 – 25.2)		**Asn/Asn**	8.5 ± 7.1<br>6.1 (2.0 – 23.8)		**G/G**	9.0 ± 7.4<br>6.4 (1.9 – 25.1)	
	**G/A**	7.8 ± 6.1<br>6.0 (1.9 – 22.7)	0.35<br>-0.36 (0.41)	**Asn/Ser**	7.6 ± 6.3<br>5.5 (1.7 – 20.4)	0.59<br>0.17 (0.33)	**G/T**	6.9 ± 5.5<br>5.0 (1.8 – 20.2)	0.00011*<br>-1.49 (0.45)
	**A/A**	7.1 ± 4.8<br>5.8 (1.1 – 18.3)		**Ser/Ser**	9.9 ± 8.3<br>7.0 (1.8 – 30.6)		**T/T**	4.0 ± 3.1<br>3.0 (0.7 – 10.5)	
**LH (IU/L)**	**G/G**	4.7 ± 2.4<br>4.2 (1.7 – 9.0)		**Asn/Asn**	4.8 ± 2.3<br>4.4 (1.7 – 9.0)		**G/G**	4.7 ± 1.8<br>4.6 (2.7 – 8.2)	
	**G/A**	4.5 ± 1.9<br>4.3 (1.8 – 7.6)	0.80<br>0.04 (0.18)	**Asn/Ser**	4.4 ± 1.9<br>3.9 (1.6 – 7.6)	0.86<br>0.02 (0.14)	**G/T**	4.5 ± 1.9<br>4.2 (1.4 – 8.1)	0.87<br>-0.03 (0.20)
	**A/A**	4.9 ± 1.4<br>5.0 (2.7 – 7.0)		**Ser/Ser**	5.1 ± 2.6<br>4.3 (2.1 – 9.1)		**T/T**	4.7 ± 1.8<br>4.6 (2.7 – 8.2)	
**Inhibin B (pg/mL)[c]**	**G/G**	81.4 ± 52.6<br>73.8 (12.7 – 169.2)		**Asn/Asn**	82.5 ± 46.6<br>79.1 (10.0 – 172.1)		**G/G**	57.6 ± 14.8<br>57.9 (43.7 – 70.8)	
	**G/A**	86.1 ± 55.7<br>76.9 (10.0 – 187.7)	0.91<br>0.86 (7.77)	**Asn/Ser**	93.8 ± 66.5<br>76.9 (24.3 – 206.6)	0.09<br>-9.36 (6.28)	**G/T**	78.5 ± 43.8<br>74.3 (10.6 – 176.9)	0.56<br>-4.56 (8.56)
	**A/A**	136.7 ± 148.3<br>68.3 (15.6 – 374.5)		**Ser/Ser**	58.1 ± 47.0<br>48.5 (10.0 – 173.2)		**T/T**	57.6 ± 14.8<br>57.9 (43.7 – 70.8)	
**Total testosterone (nmol/L)**	**G/G**	18.6 ± 6.2<br>17.7 (10.4 – 29.8)		**Asn/Asn**	19.7 ± 7.2<br>18.5 (10.3 – 32.9)		**G/G**	18.4 ± 6.7<br>17.7 (9.3 – 31.5)	
	**G/A**	18.8 ± 8.0<br>17.9 (8.1 – 35.9)	0.21<br>-0.69 (0.58)	**Asn/Ser**	17.6 ± 6.4<br>17.0 (9.1 – 30.0)	0.14<br>-0.66 (0.46)	**G/T**	19.2 ± 7.2<br>17.6 (10.2 – 32.1)	0.32<br>0.65 (0.64)
	**A/A**	16.0 ± 4.2<br>16.1 (10.0 – 26.6)		**Ser/Ser**	18.9 ± 7.0<br>17.8 (9.5 – 32.3)		**T/T**	18.7 ± 5.0<br>18.5 (10.5 – 27.1)	

**Estradiol (pmol/L)**	**G/G**	101.4 ± 37.2<br>91.0 (73.0 – 169.0)		**Asn/Asn**	106.0 ± 45.7<br>90.3 (73.0 – 179.8)		**G/G**	103.8 ± 42.3<br>91.4 (73.0 – 174.5)	
	**G/A**	104.1 ± 43.4<br>90.5 (73.0 – 173.6)	0.87<br>-0.44 (2.80)	**Asn/Ser**	99.2 ± 35.5<br>87.0 (73.0 – 155.6)	0.48<br>-1.55 (2.22)	**G/T**	96.4 ± 27.1<br>86.5 (73.0 – 153.1)	0.45<br>-2.27 (3.07)
	**A/A**	91.7 ± 29.6<br>73.4 (73.0 – 177.0)		**Ser/Ser**	101.7 ± 34.3<br>93.0 (73.0 – 197.0)		**T/T**	108.6 ± 49.3<br>78.9 (73.0 – 201.0)	
**Total testes volume (mL)**	**G/G**	38.4 ± 9.3<br>39.0 (22.0 – 52.0)		**Asn/Asn**	40.7 ± 11.0<br>42.0 (24.0 – 53.8)		**G/G**	39.3 ± 10.4<br>40.0 (22.0 – 52.5)	
	**G/A**	41.1 ± 11.3<br>41.0 (24.2 – 60.0)	0.043<br>1.82 (0.90)	**Asn/Ser**	39.2 ± 9.6<br>40.0 (22.0 – 55.5)	0.023<br>-1.63 (0.71)	**G/T**	40.3 ± 9.3<br>40.0 (25.1 – 59.7)	0.68<br>-0.42 (1.00)
	**A/A**	38.9 ± 9.8<br>36.0 (20.0 – 56.0)		**Ser/Ser**	37.4 ± 9.7<br>36.0 (22.0 – 52.9)		**T/T**	32.1 ± 7.8<br>32.5 (22.0 – 47.0)	
**Semen volume (mL)**	**G/G**	3.8 ± 1.7<br>3.6 (1.2 – 7.0)		**Asn/Asn**	3.9 ± 1.7<br>3.6 (1.5 – 7.0)		**G/G**	3.7 ± 1.6<br>3.5 (1.3 – 6.9)	
	**G/A**	3.5 ± 1.6<br>3.3 (1.3 – 6.9)	0.17<br>-0.19 (0.15)	**Asn/Ser**	3.7 ± 1.7<br>3.5 (1.3 – 7.1)	0.14<br>-0.17 (0.12)	**G/T**	3.6 ± 1.7<br>3.3 (1.1 – 7.0)	0.803<br>0.03 (0.17)
	**A/A**	3.8 ± 2.2<br>3.2 (1.3 – 9.4)		**Ser/Ser**	3.5 ± 1.5<br>3.2 (1.1 – 5.7)		**T/T**	5.1 ± 1.8<br>5.1 (2.6 – 7.8)	
**Sperm concentration (10^6/mL)**	**G/G**	4.4 ± 4.0<br>3.5 (0.1 – 11.8)		**Asn/Asn**	4.5 ± 3.9<br>3.6 (0.1 – 11.8)		**G/G**	4.2 ± 3.9<br>3.5 (0.1 – 12.0)	
	**G/A**	4.2 ± 3.7<br>3.0 (0.1 – 11.0)	0.95<br>0.02 (0.31)	**Asn/Ser**	4.2 ± 3.7<br>3.5 (0.1 – 11.4)	0.63<br>-0.11 (0.25)	**G/T**	4.8 ± 4.2<br>3.2 (0.2 – 13.0)	0.17<br>0.48 (0.35)
	**A/A**	6.1 ± 6.0<br>6.0 (0.0 – 17.6)		**Ser/Ser**	4.8 ± 5.0<br>3.0 (0.1 – 18.0)		**T/T**	4.6 ± 4.0<br>3.3 (0.2 – 11.0)	
**Total sperm count (10^6)**	**G/G**	14.2 ± 11.3<br>12.0 (0.2 – 34.5)		**Asn/Asn**	14.9 ± 11.4<br>13.8 (0.2 – 34.2)		**G/G**	13.6 ± 11.3<br>11.2 (0.2 – 34.3)	
	**G/A**	13.4 ± 10.8<br>11.8 (0.2 – 33.3)	0.69<br>-0.38 (1.01)	**Asn/Ser**	13.5 ± 11.0<br>11.0 (0.2 – 34.3)	0.25<br>-0.86 (0.81)	**G/T**	14.7 ± 10.8<br>12.3 (0.6 – 34.2)	0.108<br>1.93 (1.15)
	**A/A**	16.7 ± 13.8<br>9.5 (0.1 – 38.1)		**Ser/Ser**	13.3 ± 11.6<br>10.6 (0.3 – 34.9)		**T/T**	18.9 ± 13.0<br>19.6 (0.9 – 36.0)	

[a]Data presented as mean ± SD and median (5-95th percentile)

[b]*FSHR* -29 A-, *FSHR* 680Ser- and *FSHB* -211 T-allele effects are shown as the estimated linear regression (additive model) statistic β, standard error of the regression (SE) is shown in brackets. Asterisk (*) indicates a significant association, *P*<0.05 after Bonferroni correction for multiple testing.

[c]Inhibin B measurements were available for 159 individuals
